# 46,XX ovotesticular disorder in a Mexican patient with Beckwith–Wiedemann syndrome: a case report

**DOI:** 10.1186/1752-1947-6-301

**Published:** 2012-09-13

**Authors:** Nelly Margarita Macías-Gómez, Evelia Leal-Ugarte, Melva Gutiérrez-Angulo, Guadalupe Domínguez-Quezada, Horacio Rivera, Patricio Barros-Núñez

**Affiliations:** 1 Departamento de Salud y Bienestar, Centro Universitario del Sur, U de G. Av. Prolongación Colón s/n, Km. 1. Carretera Cd. Guzmán-Guadalajara, Ciudad Guzmán, Jalisco, 49000, Mexico; 2 Unidad Académica de Ciencias de la Salud y Tecnología, UAT, Matamoros, Tamps, Mexico; 3 Centro Universitario de Los Altos, U de G, Tepatitlán de Morelos, Jalisco, Mexico; 4 Centro de Investigación Biomédica de Occidente-IMSS, Guadalajara, Jalisco, Mexico

## Abstract

**Introduction:**

Beckwith–Wiedemann syndrome is an overgrowth syndrome that is characterized by hypoglycemia at birth, coarse face, hemihypertrophy and an increased risk to develop embryonal tumors. In approximately 15% of patients, the inheritance is autosomal dominant with variable expressivity and incomplete penetrance, whereas the remainder of Beckwith–Wiedemann syndrome cases are sporadic. Beckwith–Wiedemann syndrome molecular etiologies are complex and involve the two imprinting centers 1 (IC1) and 2 (IC2) of 11p15 region. This case report describes, for the first time, the unusual association of ovotesticular disorder in a patient from Morelia, Mexico with Wiedemann-Beckwith syndrome.

**Case presentation:**

We report the case of a Mexican six-year-old girl with Beckwith–Wiedemann Syndrome, ambiguous genitalia, and bilateral ovotestes. She has a 46,XX karyotype without evidence of Y-chromosome sequences detected by fluorescence in situ hybridization with both SRY and wcp-Y probes.

**Conclusion:**

Although a random association between these two conditions cannot be excluded, future analysis of this patient with Beckwith–Wiedemann syndrome and 46,XX ovotesticular disorder may lead to new insights into these complex pathologies. We speculate that a possible misregulation in the imprinted genes network has a fundamental role in the coexistence of these two disorders.

## Introduction

Beckwith–Wiedemann syndrome (BWS; Mendelian Inheritance in Man [MIM] #130650), the most common overgrowth syndrome, has been described worldwide with an incidence of 1 in 12,000 to 13,700 live births, and is characterized by macrosomia, macroglossia, visceromegaly, abdominal wall defects, hemihyperplasia, renal abnormalities, hypoglycemia and a predisposition (7% to 21%) to develop embryonic tumors in the first decade, with the highest risk before the age of 5 years [1]. BWS is a heterogeneous syndrome and its molecular etiology is very complex because many mechanisms are involved. Most cases of BWS are sporadic, however, approximately 10% to 15% of BWS cases present positive family history in which autosomal dominant transmission with variable expressivity and incomplete penetrance is demonstrable. Several studies have reported the involvement of five principal genes distributed in two imprinting control regions (ICR1 and ICR2) at the critical 11p15 BWS region in both familial and sporadic BWS. Genetic and epigenetic alterations described in BWS include chromosomal abnormalities (translocations, inversion, and duplications), paternal uniparental disomy, hypo or hypermethylation at ICR1 and ICR2 and mutations in *CDKN1C*[2,3]*.* Even though molecular studies are useful diagnostic tools for BWS, to date, all these findings only allow us to identify the molecular abnormalities involved in the 80% to 85% of patients with BWS, so a negative test cannot discard the BWS diagnosis which is still based on clinical criteria [4-8]. We report here on the novel association of BWS with 46,XX ovotesticular disorder found in a Mexican patient.

## Case presentation

**Figure 1:**
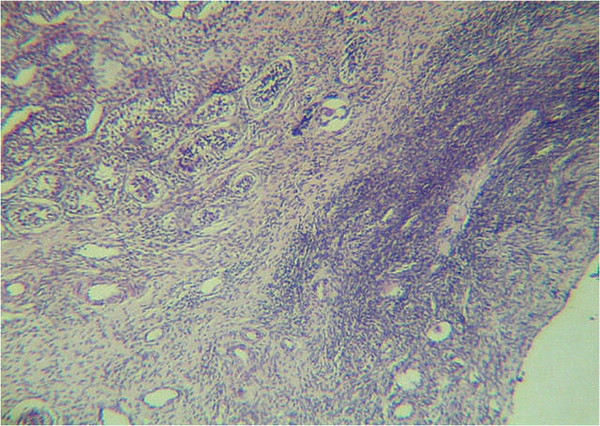
**Ovotestes.** Gonadal histology with primary follicles (right side) and immature testicular tissue (left side).

**Figure 2:**
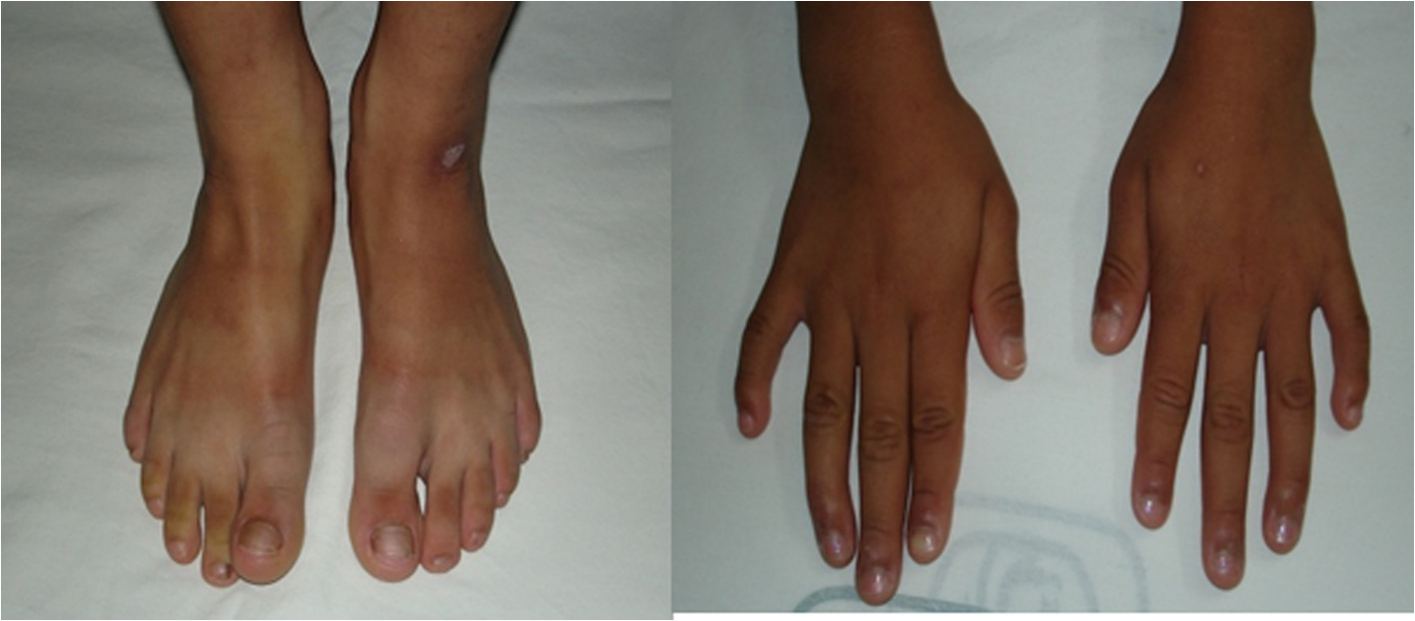
**Proband’s hands and feet.** Observe the brachydactyly, cutaneous syndactyly of second and third toes, and pigmentation abnormalities on the skin and nails.

We report the case of a Mexican six-year-old girl who was born as the fourth child of young, healthy and unrelated parents; pregnancy and family history were unremarkable. The child was delivered at the 40th week but, because of respiratory distress, forceps and reanimation were required. Neonatal information such as APGAR, weight, length, and hypoglycemic events was not recorded. At birth, umbilical and inguinal hernias as well as genital ambiguity with enlarged clitoris, undersized vaginal introitus, and hypoplastic and hyperpigmented labia were noticed. A hormonal profile and abdominal sonographic study discarded suprarenal hyperplasia or abdominal tumors. Ovotestes were demonstrated by histological studies after a laparotomy was performed to evaluate gonadal tissue and accessory sex organs (Figure 1). Psychomotor development was delayed. At six years of age, the patient exhibited weight of 23kg (90th to 97th percentile), stature of 125cm (>97th percentile), occipital-frontal circumference of 52cm (97th percentile), brachydactyly and clinodactyly of her fourth and fifth fingers, low-set hairline on neck and forehead, facial *nevus flammeus*, dysplastic and low-set ears with grooves in both lobes, coarse facial features, macroglossia, and wide neck and hemihypertrophy of all of the left side (1.5cm). In addition to ovotestes and brachydactyly and clinodactyly of her fourth and fifth fingers, the proband also showed some atypical features such as cutaneous syndactyly and multiple *café au lait* spots in all the body (Figure 2). Cytogenetic analysis was done on preparations obtained from a peripheral blood lymphocyte culture and stained for GTG-bands; her karyotype was 46,XX in 16 metaphases. In order to explain the presence of ovotestes, we looked for chromosome Y-sequences by means of fluorescence in situ hybridization (FISH) with whole chromosome painting (WCP)-Y and LSI SRY Spectrum Orange/CEP X Spectrum Green dual probe (Vysis, Illinois, USA). The scoring of >10 metaphases and >50 interphases per assay did not show Y-chromosome sequences.

## Discussion

In our patient, the BWS diagnosis was based on the typical clinical findings as well as additional features such as wide neck, micro-clinodactyly of fourth and fifth fingers and toes, skin syndactyly, skin pigmentation abnormalities and 46,XX ovotesticular disorder. To the best of our knowledge, this is the first BWS patient found to have a coexisting ovotesticular disorder. Because gonadal differentiation involves a very complex genetic program at different embryonic stages, the occurrence of any disorder of sexual development (DSD) is a clinical challenge. The ovotesticular disorder is a rare DSD and the predominant karyotype (70% of cases) in these patients is 46,XX followed by chimerism XX/XY in about 20% [9]. Testicular differentiation is accounted for by the presence of the master *SRY* gene in only 2% to 12% of cases, and remains unexplained in the remainder of cases. However, it has been suggested that mutations or deregulation of other genes involved in gonadal development in a 46,XX embryo can produce ambiguous genitalia [10,11]. One marker of mammalian testis development is the size increase of rudimentary XY gonad, which is determined by the presence of *SRY.* The male-size increase is the result of coelomic cell proliferation after 24 hours of *SRY* gene expression, and originates in two stages. The first stage is the proliferation of SF1-positive cells, which will eventually give rise to the Sertoli cells. The second stage is the proliferation of SF1-negative cells. It has been postulated that this cell proliferation alters the transcriptional accessibility of the *HOX* gene clusters, thereby controlling the timing of the expression of specific genes and consequent cell differentiation [12]. In the present patient we did not find any Y-chromosome sequences by FISH; however, we cannot rule out the possibility of a cryptic mutation or a small Y-bearing clone. Moreover, our patient also shares features with the Denys–Drash syndrome (Online Mendelian Inheritance in Man [OMIM] #194080) mapped in 11p13 whose distinctive features are Wilms’ tumor and a DSD similar to the 46,XX ovotesticular disorder. The implicated gene in Denys–Drash syndrome is *WT1* which encodes for a zinc finger transcription factor that is expressed very early in the urogenital ridge and plays a crucial role in gonadal differentiation. A concurrent mutation in the *WT1* gene may explain the DSD in our patient. Recent reports in mice indicate the imprinted genes network (IGN) regulates the gene expression through modulation of the imprinting pattern at different embryogenesis stages [13,14]. For instance, the transcription factor *ZAC1*, one of the major IGN genes, especially modulates the expression of *H19* and/or *IGF2* genes. Accordingly, we speculate that in our patient a change in the methylation pattern of *ZAC1* modifies the expression of *H19* and/or *IGF2* and gives rise to BWS phenotype, and perhaps even to the brachydactyly, because such a gene is highly expressed in chondrogenic tissue [15]. Because *ZAC1* also regulates *SOX11,* whose possible function is in gonadal development [16], the patient’s 46,XX ovotesticular disorder could result from an impaired IGN, although a random association cannot be excluded. In order to clarify the presence of these two complex pathologies (BWS and 46,XX ovotesticular disorder) in our patient is important to carry out future molecular analysis.

## Conclusion

We report an unusual clinical presentation of BWS including a 46,XX ovotesticular disorder in a Mexican girl. Although a random association among these two conditions cannot be excluded, the coexistence of BWS and 46,XX ovotesticular disorder may lead to new insights in gonadal development. We speculate that a possible deregulation of the IGN has a fundamental role in the coexistence of these two disorders. To the best of our knowledge this is the first case where these two pathologies are coexisting.

## Consent

Written informed consent was obtained from the patient’s legal guardian for publication of this manuscript and accompanying images. A copy of the written consent is available for review by the Editor-in-Chief of this journal.

## Competing interests

The authors declare that they have no competing interests.

## Authors’ contributions

Our patient was admitted and followed in-house by BNP, MGN and LUE. GAM, RH and DQG performed the laboratorial test. MGN and RH performed the major contribution in writing the manuscript. All authors read and approved the final manuscript.
